# Prevalence and correlates of hypertension in HIV-positive adults from the Livingstone Central Hospital, Zambia

**DOI:** 10.11604/pamj.2021.39.237.29718

**Published:** 2021-08-12

**Authors:** Robert Musekwa, Benson Malambo Hamooya, John Robert Koethe, Selestine Nzala, Sepiso Kenias Masenga

**Affiliations:** 1 School of Medicine and Health Sciences, Mulungushi University, Hand Research Group, Livingstone, Zambia,; 2 Vanderbilt University Medical Center, Nashville, Tennesse United States of America,; 3 University of Zambia, School of Medicine, Lusaka, Zambia

**Keywords:** Hypertension, HIV, dolutegravir, antiretroviral therapy, body mass index, age

## Abstract

**Introduction:**

HIV-infection and treatment with antiretroviral therapy (ART) are risk factors for the development of hypertension, which is more prevalent in people living with HIV compared with the general population. Although there is a shift to Integrase Strand Transfer Inhibitor (INSTI)-based ART across the sub-Saharan Africa, there is limited information with regard to INSTIs and hypertension association in this region, making this, a critical question to address. Hence, the study aimed to determine the relationship between hypertension and ART regimen in people living with HIV.

**Methods:**

this was a cross-sectional study conducted at the Livingstone Central Hospital, southern province of Zambia. This study utilized programmatic data. Demographic and clinical data of 348 persons living with HIV who had been on ART for more than 2 years was abstracted in the adult ART database registry. Descriptive and inferential statistics were used for analyses of data.

**Results:**

prevalence of hypertension was 18.4% (n=64). Hypertensives were older than normotensives with median (interquartile range) age of 55 (49, 61) and 46 (41, 52), respectively. At multivariate analysis, age (aOR: 1.07, 95% CI 1.04-1.11; p = 0.001) and body mass index (aOR: 1.10, 95% CI 1.04-1.16; p = 0.002) were positively associated with hypertension. Participants on dolutegravir based regimen were 2 times (aOR: 2.44, 95% CI 1.22-4.86; p = 0.01) more likely to be hypertensive compared to those on non-nucleoside reverse transcriptase inhibitors (efavirenz or nevirapine).

**Conclusion:**

we confirm that increasing age, body mass index (BMI) and use of dolutegravir are risk factors for hypertension. Close monitoring for persons with HIV with these known risk factors is required.

## Introduction

Hypertension is more common among people living with HIV (PWH) compared with the general population, and the use of antiretroviral therapy (ART) has been identified as one of the underlying factors contributing to the development of hypertension [1-3]. Newer classes of ART introduced in Zambia, including integrase strand transfer inhibitors (INSTIs) and protease inhibitors (PIs) have been associated with incidence and development of metabolic derangements and weight gain, which are linked to the genesis and progression of hypertension in some studies [4-6]. In sub-Saharan Africa (SSA) there is a widespread adoption of INSTI-based regimens, specifically dolutegravir (DTG), but there is limited information on the association between DTG and hypertension in this region. Hence, the goal of our study was to compare the prevalence of hypertension among PWH on different commonly used ART regimens in Zambia.

## Methods

**Study design and setting:** we conducted a cross-sectional study using programmatic data at Livingstone Central Hospital (LCH) ART clinic. LCH is the largest referral hospital in Southern Province of Zambia that offers ART and general medical services to the community with approximately 3700 - 4000 PWH enrolled in ART care.

**Study population:** this study included normotensive and hypertensive adult PWH who had been on ART for more than 2 years. Participants with incomplete data on more than 2 blood pressure readings per year, enrolment date, age, and ART specific regimens were excluded from the study. OpenEpi, Version 3, an online open-source calculator program found at openEpi.com was used to calculate maximum sample size of 348 for an estimated 3800 PWH enrolled in the ART clinic at 80% power and 95% CI. The hypothesized prevalence of hypertension we used was 50% to maximize power of detection. Participants were selected using systematic sampling. We divided the sample size into the population that met the inclusion criteria (3800/348) and sampled every 10^th^ patient in the records using an interphase in the ART database registry that systematically selects PWH on ART.

**Data collection:** a data collection form was used to abstract data from HIV care summary sheets and smartcare patient summary reports. The primary outcome was hypertension, dichotomized as “normotensive” and “hypertensive”. The independent variables abstracted were age, gender, ART regimen class, BMI, creatinine, switching of ART in the course of treatment, current CD4 count, CD4 count at initiation and viral load. Viral suppression was defined by a viral load of less than 1000 copies/ml. Hypertension status was determined by a systolic and diastolic blood pressure (SBP/DBP) of equal to or higher than 140/90mmHg on more than 2 occasions during routine visits or history of using antihypertensive drugs on file. The INSTIs and protease inhibitors (PIs) participants used was dolutegravir and ritonavir boosted lopinavir based regimen, respectively. The non-nucleoside reverse transcriptase inhibitors (NNRTIs) were efavirenz (EFV) and Nevirapine (NVP).

**Statistical analysis:** we used descriptive statistics such as means, medians and frequencies to describe the data. For inferential statistics, we used Mann Whitney test to compare medians of all continuous explanatory variables between hypertensives and normotensives and a chi-square to test associations between categorical variables. Multivariate logistic regression model was used to determine factors associated with hypertension and to control for confounding. All variables with a p-value less than 0.02 in the univariate analysis were included in the multivariate logistic regression model to avoid overadjustment. P-value of less than 0.05 was considered to be statistically significant at 95% confidence Interval. For all data analysis Statistical Package for Social Sciences (SPSS) version 22 and GraphPad prism version 9 were used.

**Ethical considerations:** ethical approval was obtained from the Mulungushi University School of Medicine and Health Sciences Research Ethics Committee (IRB: 00012281 FWA: 0002888) on 10^th^ March 2020. Permission to conduct the study was granted by Livingstone Central Hospital Administration. Data collected was de-identified and used for research purposes only.

## Results

**General characteristics:** the study comprised 348 participants with median age (interquartile range) 47 years (42, 55) and 221 (63.5%) were females. About 59%, 26% and 15% were on NNRTIs, INSTI and PI-based regimens, respectively. Hypertensive participants had a higher median BMI (26 vs 22) and were older (55 vs 46) compared to the normotensive, p = 0.001. Among participants on dolutegravir, a significantly higher proportion (47%) were hypertensive compared to normotensive (21%). Among participants who switched ART in the course of treatment and virally suppressed, a significantly higher proportion was hypertensive (94% and 98%, respectively) compared to the normotensive (81% and 83%, respectively), p=0.05 (Table 1).

**Table 1:** factors associated with hypertension

Variable	Hypertension status, n (%)			
	**Hypertensive, 64 (18.4)**	**Normotensive, 284 (81.6)**	**Total, n (%)**	**p-value**
**Age, median years (IQR)**	55 (49, 61)	46 (41, 52)	47 (42,55)	**<0.0001**
**Gender**				
Male	28 (43.8)	99 (34.9)	127 (36.4)	0.18
Female	36 (56.3)	185 (65.1)	221 (63.2)	
**ART regimen**				
NNRTI (EFV or NVP)	25 (39.1)	181 (63.7)	206 (69.2)	**0.004**
INSTI (DTG)	30 (46.9)	59 (20.8)	89 (25.6)	
PI (LPV/r)	9 (14.1)	44 (15.5)	53 (15.2)	
**Switched ART regimen**				
Yes	60 (93.8)	230 (81.0)	290 (83.3)	0.015*
No	4 (6.3)	54 (9.0)	58 (16.7)	
**Body mass index**	26.1 (21.4, 30.0)	22.2 (19.5, 26.1)	22.7 (19.7, 27.2)	**<0.001**
Current CD4 Count, cells/μL	472 (335, 617)	463 (334, 660)	461(334, 621)	0.71
CD4 at initiation, cells/μL	162 (112, 294)	195 (91, 305)	185 (94,297)	0.72
**Viral suppression <1000 copies/ml**				
Yes	53 (98.1)	180 (83.3)	233 (100)	0.003*
No	1 (1.9)	36 (16.7)	37 (100)	
**Creatinine**, μmol/L	82 (67, 101)	74 (59, 91)	75(60,91)	0.06

NNRTI=Non-Nucleoside Reverse Transcriptase Inhibitor; NRTI=Nucleoside Reverse Transcriptase Inhibitor; DTG= dolutegravir; LPV/r= ritonavir boosted lopinavir; IQR=interquartile range; *fisher's exact test

**Prevalence and correlates of hypertension:** the prevalence of hypertension was 18%. Age (aOR: 1.07, 95% CI 1.04-1.11; p = 0.001) and BMI (aOR: 1.10, 95% CI 1.04-1.16; p = 0.002) were positively associated with hypertension on multivariate analysis (Table 2). Participants on dolutegravir based regimen were 2 times more likely to be hypertensive compared to those on NNRTI/NRTI based regimen on multivariate analysis (aOR: 2.44, 95% CI 1.22-4.86; p = 0.01). Use of protease inhibitors was not associated with hypertension (aOR: 1.58, 95% CI 0.63-3.97; p = 0.21). On univariate analysis, PWH who Switched ART in the course of treatment were about 4 times more likely to be hypertensive compared to those who did not switch ART (OR: 3.52, 95% CI 1.23-10.11; p = 0.019). However, this association did not remain significant at multivariate analysis (aOR: 2.06, 95% CI 0.66-6.47; p = 0.21).

**Table 2:** factors associated with hypertension in logistic regression

Variable	Univariable analysis		Multivariable analysis	
	**OR (95% CI)**	**p-value**	**aOR (95% CI)**	**p-value**
**Age, median years (IQR)**	1.0 (1.0, 1.1)	<0.001	1.07 (1.04, 1.11)	<0.001
**Gender**				
Male	1			
Female	0.6 (0.4, 1.2)	0.18		
**ART regimen**				
NNRTI/NRTI	1		1	
Integrase (DTG) based	3.7 (2.0, 6.7)	<0.001	2.44 (1.22, 4.86)	0.011
Protease (LPV/r) inhibitor based	1.4 (0.6, 3.4)	0.35	1.58 (0.63, 3.97)	0.21
**Switched ART regimen**				
No	1			
Yes	3.52 (1.23, 10.11)	0.019	2.06 (0.66, 6.47)	0.21
**Body mass index**	1.1 (1.0, 1.2)	<0.001	1.10 (1.04, 1.16)	0.002
**Viral suppression <1000 copies/ml**				
Yes	1			
No	0.09 (0.01, 0.70)	0.021		
**Current CD4 Count**, cells/μL	1.0 (1.0, 1.0)	0.32		
**CD4 at initiation**, cells/μL	1.0 (0.9, 1.0)	0.83		
**Creatinine**, μmol/L	1.0 (0.9, 1.0)	0.67		

CI=confidence interval; aOR= adjusted odds ratio; OR= Unadjusted odds ratio; NNRTI=Non-Nucleoside Reverse Transcriptase Inhibitor; NRTI=Nucleoside Reverse Transcriptase Inhibitor; DTG= dolutegravir; LPV/r= ritonavir boosted lopinavir; IQR=interquartile range

## Discussion

Our study consisted of 348 PWH who had been on ART for more than 2 years. Participants were either on NNRTI (EFV or NVP), INSTI (DTG) or protease inhibitors (PI) (LPV/r). We found that hypertension was prevalent (18%) among our study participants, similar to what has been reported before in sub-Saharan Africa [2]. Hypertension in PWH has greatly increased in the previous years and has been highly associated with the increase in cardiovascular morbidity and mortality [7-9]. Compared to HIV negative individuals, HIV positive individuals on ART are more susceptible to the development of hypertension [10]. Several virologic and treatment-related factors are believed to play a role in the pathophysiology of hypertension in HIV infection, including chronic inflammation, immune reconstitution, and lipodystrophy, of which, all seem to directly or indirectly influence common downstream pathways such as the sympathetic and renin-angiotensin-aldosterone systems (RAAS) [10]. We found that increasing age and BMI was significantly associated with hypertension. These findings coincide with previous reports [5,11,12]. It is a known fact, held for many decades that increasing age and BMI are independent predictors of hypertension. We also found that use of the INSTI dolutegravir was a significant predictor of hypertension. In literature, use of specific ART including dolutegravir to treat HIV has also been associated with altered lipid metabolism, weight gain and accelerated atherosclerosis which increases blood pressure in PWH [5,6,13,14]. The mechanism of dolutegravir in increasing weight of PWH and the risk of hypertension is unknown. However, it is thought that INSTIs like dolutegravir interfere with the hypothalamic control of food intake and energy homeostasis through the melanocortin signaling system, the most potent physiological control mechanism of food intake behavior and metabolic energy balance in mammals [15]. In addition, dolutegravir is also associated with altered adipose tissue distribution and deposition characterized by increased inflammation in HIV and obesity [5]. Other factors that synergize to increase the risk for and exacerbate existing hypertension includes but not limited to increasing age, immune-activation and level of viremia, obesity, low education, sedentary lifestyle, smoking, family history, alcohol use, and diet [2,16,17].

**Figure 1:**
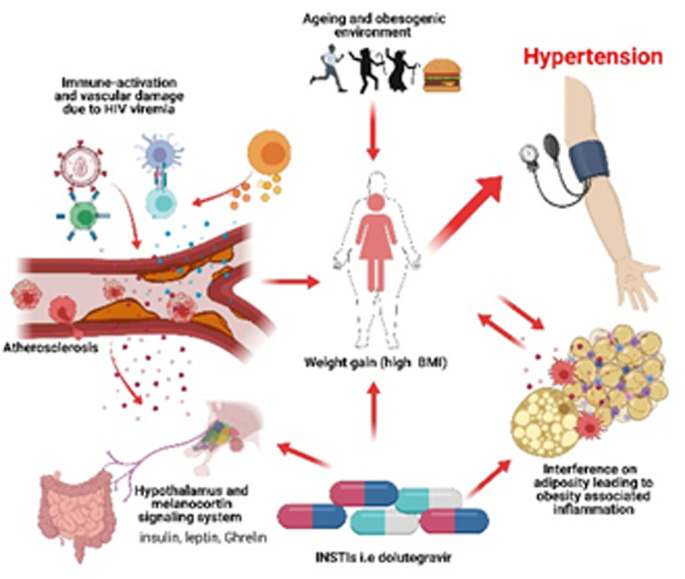
illustration of factors contributing to the genesis and progression of hypertension

Although an association between low nadir CD4 cell count and increased blood pressure (BP) after initiation of ART has been observed in some studies [8,18], both current and nadir CD4 was not associated with hypertension in our study. Immuno-suppression plays a role in promoting early vascular damage as evidenced by the association of a low CD4 cell count with subclinical atherosclerotic damage [19]. We found that participants without HIV viral suppression were at lower risk of developing hypertension compared with virally suppressed PWH. However, evidence regarding the association between HIV viral load and hypertension is inconsistent in literature and using viremia copy-years, a cumulative measure of HIV plasma viral burden is more effective than single time-point viral load measures [20]. We hypothesize that in HIV, specific antiretroviral therapy (ART) such as integrase strand transfer inhibitor (INSTIs) based regimen like dolutegravir alter the melanocortin signaling system and adipose distribution (Figure 1). This results in weight gain (evidenced by high BMI), associated with inflammation and damage to vasculature in the face of HIV RNA viral suppression and ageing characteristics that synergize in contributing to the development of hypertension. As evidenced from literature, the underlying rationale for developing hypertension may involve HIV viral load that may cause damage to cells and the vasculature [21]. In addition, production of inflammatory cytokines elicited by cell death and viral replication may directly and indirectly injure the vasculature and kidneys [21,22]. Moreover, cells of the innate immunity produce reactive oxygen species causing oxidative stress and leading to endothelial dysfunction. Endothelial dysfunction disrupts nitric oxide availability leading to constriction of vessels and in this way contributing to hypertension [21]. However, in PWH, the role of INSTIs in contributing to hypertension, in addition to an obesogenic environment (high-fat diet and physical inactivity), shifting demographics, and an aging may dominate, resulting in increased BMI associated with altered adiposity and increased inflammation. The strength of this study, based on the use of available secondary data to understand the possible interaction or relationship between hypertension and ART regimen and other demographic and clinical characteristics of clinical interest was an advantage and suitable for hypothesis generation. However, because hypertension development has multifactorial underlying factors, we are likely to miss many factors that may contribute to hypertension with the usage of secondary data. Hence, further studies are warrantable.

## Conclusion

We confirm that age, BMI and use of INSTI dolutegravir are predictors of hypertension. Close monitoring for persons with HIV with these known risk factors is required.

**Funding:** this work was supported by the Fogarty International Center of the National Institutes of Health (D43 TW009337 and D43 TW009744). The content is solely the responsibility of the authors and does not necessarily represent the official views of the National Institutes of Health.

### What is known about this topic

- Dolutegravir is associated with weight gain;
- Body mass index and increasing age are associated with hypertension.

### What this study adds

- Dolutegravir is associated with hypertension in a younger Zambian population of black persons living with HIV independent of age, body mass index and viral suppression.
